# Thigh gaps and filtered snaps: a qualitative study exploring opportunities to mitigate social media harm through content moderation for people with eating disorders

**DOI:** 10.1186/s40337-025-01504-7

**Published:** 2026-01-21

**Authors:** Pranita Shrestha, Jue Xie, Pari Delir Haghighi, Michelle L. Byrne, Scott Griffiths, Roisin McNaney

**Affiliations:** 1 Department of Human-Centred Computing, Monash University, Wellington Rd, Clayton, VIC 3800 Australia; 2 School of Psychological Sciences, Turner Institute for Brain and Mental Health, Building 1/270 Ferntree Gully Rd, Notting Hill, VIC 3168 Australia; 3 Melbourne School of Psychological Sciences, University of Melbourne, Barry Building, Redmond, Parkville, VIC 3010 Australia; 4 School of Computing and Information Systems, University of Melbourne, Carlton, VIC 3052 Australia

**Keywords:** Social media, Body dissatisfaction, Eating disorders, Social media content

## Abstract

**Background:**

The ubiquity of social media has increased exposure to idealised beauty standards, often unrealistic and harmful. Repeated exposure has been linked to body dissatisfaction, harmful behaviours, and potentially the development of eating disorders (ED). Given the volume of content produced daily, effective harm mitigation strategies (automated or user-driven) are essential, requiring an informed understanding of the contexts and nuances surrounding harmful content.

**Objective:**

The study has two key aims: (1) to understand the perspectives of experts by profession and people with lived experience of ED, on what makes social media content harmful in the context of body image and ED, including why and how this harm occurs; and (2) to explore how technology might help mitigate these effects.

**Methods:**

We engaged *n* = 30 participants, including 12 interviews with experts by profession (*n* = 2 ED support service providers and *n* = 10 body image and ED experts), and five focus groups with experts by lived experience (*n* = 18 people with lived experience of ED).

**Results:**

Using the Framework Method guided by inductive thematic analysis, we developed six prominent themes: (1) Spectrum of harmful and ambiguous content on social media, (2) The “echo chamber” of harmful content amplified by social media algorithms, (3) Balancing safety, freedom and responsibility in social media moderation, (4) Shared responsibility and collaboration for safer social media environments, (5) The role of representation and diversity in social media recovery and support, and (6) Harnessing digital innovation to reduce harm on social media. We developed an eight-category framework of harmful social media content, offering an underlying contextual understanding of harmful content and guidance for harm-reducing technologies.

**Conclusions:**

Manual safeguards place significant responsibility on users. This work supports informed distinctions between harmful, ambiguous and safe content and provides design insights for classification systems and adaptable automated moderation.

**Supplementary Information:**

The online version contains supplementary material available at 10.1186/s40337-025-01504-7.

## Abstract (plain language summary)

Constant exposure to idealised beauty standards online can lead to negative body image, unhealthy behaviours and eating disorders (ED). To reduce these harms, we need effective ways to identify and moderate harmful content. However, first, we must understand why and how this content causes harm. We consulted *n* = 30 participants, including interviews with *n* = 12 professionals in ED support and research, and five focus groups with *n* = 18 individuals with lived experience of ED. Six major themes were developed using the Framework Method guided by inductive thematic analysis: the range of harmful and ambiguous content, social media’s contribution to an ED “echo chamber,” challenges in content moderation, the need for shared responsibility in creating safer spaces, the importance of diverse representation and the potential for technology to help mitigate harm. From this analysis, we developed eight categories of harmful social media content related to body image and ED, clarifying how individuals may be negatively affected and providing structure for future interventions. Participants highlighted the powerful influence of algorithms in promoting harmful content and called for shared responsibility among users, content creators, platforms, and policymakers. The findings offer guidance for designing technologies that mitigate social media harm.

**Supplementary Information:**

The online version contains supplementary material available at 10.1186/s40337-025-01504-7.

## Introduction

### Background

 Each year, approximately 3.3 million individuals are affected by the physical and mental impact of eating disorders (ED) such as Anorexia Nervosa, Bulimia Nervosa and Binge Eating Disorder [1]. EDs are closely connected with body image concerns; negative thoughts, perceptions, feelings, and emotions regarding appearance, shape, size, and other physical attributes [2]. Individuals with negative body image can often develop disordered eating behaviours (chronic dieting, purging calories through dieting, skipping meals, or obsessive calorie counting), which in turn can lead to the development of ED [3].

Online communities that encourage and normalise disordered eating behaviours and ED, especially Anorexia Nervosa, have been around since the 1990s in the form of websites and discussion forums [4, 5]. Over time, these online communities have persisted and transitioned from websites and discussion forums to more visually engaging social media platforms [5]. Research has shown that content from image-centric and video-centric social media platforms such as Instagram and TikTok has a strong association with ED pathology [6, 7]. These platforms prioritise appearance-based content [8], often showcasing highly idealised [9], before-and-after transformations [10] and digitally altered bodies [11, 12]. Some content emphasises restrictive eating practices such as limiting food groups, counting calories or promoting very low-calorie meals as well as excessive exercise routines to achieve a particular body shape [13]. Exposure to such material can normalise unhealthy behaviours, contributing directly to the development or exacerbation of ED pathology.

Prime examples of social media’s influence on negative body image are the rise of online ‘challenges’ (e.g. the thigh gap challenge, where women strive to achieve a noticeable gap between their inner thighs when standing with their feet together [14]) and the popularisation of weight loss drugs (e.g. Ozempic), endorsed by celebrities and content creators on social media [15]. However, harm is not always encased in such explicit examples. Research has shown that adolescents frequently use social media as a primary source of information about diet, nutrition and fitness. This often comes from creators who are not credible professionals, and can often promote unhealthy behaviors within the framing of a healthy lifestyle (e.g. eating an unbalanced diet by cutting out food groups such as carbohydrates, demonisation of sugar, fasting) [16]. A further issue is the fact that this type of harmful content is frequently amplified by social media algorithms, which recommend similar content and reinforce similar messaging to the individual [17]. With numerous research studies finding a clear connection between viewing this type of content on social media, body dissatisfaction and increased disordered eating behaviours [16, 18], this causes even more of a challenge for vulnerable users.

Given the danger and prevalence of such harmful influences, effective content moderation has become crucial to creating a safer online environment. Content moderation is the monitoring and identification of harmful content that goes against legal, ethical and community standards and taking the necessary steps towards removing such content [19, 20]. Most popular platforms (e.g. Facebook [21], Instagram [22], TikTok [23]) each have a version of automated content moderation processes [24–26]. These platforms use Artificial Intelligence (AI) algorithms to analyse visual and audio content and attached textual data (keyword, hashtag, title, caption) to determine its safety [24–26]. If content is deemed unsafe, it is either entirely removed or its spread is limited (e.g. not shown to users under 18). For example, Meta’s community standards strictly mention the removal of content including child abuse, nudity, suicide and self-harm, explicit ED content, and hate speech [27]. Whilst this is a necessary first step in keeping social media communities safe, there is a distinct lack of transparency in the algorithms that make these decisions, and often only the most extreme content is flagged.

To supplement automated AI-driven content moderation efforts, many social media platforms offer options for self-moderation, wherein the users themselves proactively moderate and regulate the kind of content they interact with through reporting and flagging. Once content has been flagged, human moderators from the specific platform review the content and make a final decision regarding whether the content breaches community guidelines [24–26]. Platforms also provide options to users to unfollow, mute and block certain content to enhance their ability to curate and moderate their feeds. However, research has shown that users often do not have sufficient critical thinking skills to enable them to identify harmful content [28]. This is particularly challenging in the context of ED development, as harmful behaviours related to disordered eating and body image concerns can often emerge gradually and may go unrecognised, concealed or misinterpreted by individuals, either consciously or unconsciously [29], which can lead to lasting harm.

Research has found that, despite content moderation efforts, a significant amount of ED-related content is reaching users [17, 30]. In a study by Griffiths et al. [17], the authors reported that the TikTok algorithm recommended 4343% more harmful ED content, 335% more dieting content and 142% more exercise content to people experiencing ED, in comparison to people not experiencing ED. Similarly, another study [31] reported that amongst 1000 videos recommended by the YouTube algorithm to a hypothetical 13-year-old girl (a profile created by researchers), 34.4% were considered as harmful eating disorder content and 63.8% were videos related to weight loss or ED.

Given that harmful content continues to evade existing moderation systems, it has become clear that protecting users cannot rely on platform-driven solutions alone. Research has highlighted that creating a safe social media environment requires a collective and shared commitment from individuals, content creators, human moderators, platforms and governments [32–35]. Due to the ever-growing volume of social media content, such collaborative efforts from different stakeholders can be enhanced further through automated technological systems. These can go beyond those already in place by social media platforms so as to reduce the load (on individuals, content creators and human moderators) to make content moderation choices. In the ED space, studies have explored using machine learning techniques to identify ED-related Reddit posts [36, 37]. In addition, Abuhassan et al. [38] applied machine learning techniques to differentiate users at-risk of developing ED, from users who are merely engaging with ED-related content, based on their interactions on Twitter (now X). Numerous research studies have used Natural Language Processing (NLP) (a branch of Artificial Intelligence (AI) that understands and interprets human language) [39] to understand harmful content from textual data such as captions, hashtags and keywords to identify pro-ED content [38, 39]. Feldman et al. [40] moved from just text-based analysis to incorporating both static image and text in social media content to classify harmful or safe content using Computer Vision (another branch of AI that understands and interprets visual information) [41]. The study developed an image classifier to distinguish between harmful (promoting ED) and safe (not promoting ED) content based on visual cues. As the study suggests, it is important to consider the visual aspect of social media due to the increase in the popularity of visual-based social media platforms.

However, despite these technological advances, existing systems still struggle with the contextual complexity of ED-related content that go beyond overtly obvious harmful content. Prior research has shown that it is relatively straightforward to identify extremely harmful content, such as content that provides instruction for extreme weight loss or mocking people with eating disorders [42]. However, for more ambiguous content, the context can differentiate whether the content is harmful or harmless [43]. One example of this is, “What I eat in a day” videos, where individuals provide information about their daily meals, often alongside calorie counts or portion sizes [44]. For individuals who are at-risk of or experiencing ED, this kind of content can lead to obsessive calorie tracking, food fixation and body dissatisfaction [45].

Beyond these challenges, an additional layer of complexity arises from contextual ambiguity present in multimodal content. Much of the existing research has been concentrated on identifying and categorising content on text-based social media platforms. While textual information (e.g. #proana) is relatively unambiguous in the context of content moderation, there are often contextual subtleties in multimodal content (audio, visual and text) that can cause detection issues [46]. This becomes complex when there are conflicting sources of context, the essential background information that facilitates an understanding of the particular situation [47], within a single instance of content. For example, a video might show a content creator on a treadmill, with a music-based audio track and a caption saying “strong and healthy”, yet could have a textual overlay on the video saying “72 hours only water!”. In these cases, understanding visual context and subtle nuances becomes increasingly important. The level of subjectivity surrounding visual content (i.e. understanding what might be considered harmful and for whom) and the content’s dependence on contextual nuances create a challenge in developing approaches to automation for detecting potentially harmful content [46]. Thus, it is important to understand the context of such content to understand possible underlying subjectivities and to design and develop technology to provide better content navigation and moderation.

The complexities are further compounded by an important question regarding who determines what constitutes harmful content. Given the subjectivity of harm, such judgements are often shaped by cultural, social and political values rather than objective criteria [20]. Consequently, automated moderation systems risk reinforcing dominant ideologies and marginalising minority voices. Furthermore, these systems raise significant concerns about transparency, accountability and explainability, as users are often unaware of how the moderation decisions are made within what is often perceived as a “black-box” algorithmic process [48].

Finally, a tension remains in relation to potential over-moderation. Chancellor et al. [49] stressed the importance of combining automated content moderation with alternative intervention solutions, rather than simply implementing blanket bans on certain content types. During content moderation, it is equally important to ensure that enforcement does not unintentionally suppress peer support networks or content from service providers [50]. For example, censoring recovery posts that contain specific hashtags or keywords could potentially silence individuals seeking help or sharing their recovery journey or coping mechanisms.

These challenges highlight the need for a deeper empirical understanding of how harmful and ambiguous ED-related content is interpreted and why such content may still cause harm. Our study directly addresses a critical gap in existing moderation approaches by exploring how ambiguous ED-related content can subtly reinforce harmful behaviours, despite appearing innocuous, through contextual cues. While current algorithmic systems primarily detect only the most extreme and explicit forms of ED-related content, challenges persist in the contextual nuances of ambiguous content that might appear benign, yet may be equally damaging depending on its framing, intent or the vulnerability of the viewer. Our work contributes a deeper understanding of why context-awareness is essential for moderation and offers recommendations for how future algorithmic scanning and content classification systems could better incorporate nuance, cues and user vulnerability for harmful content detection.

### Goal of this study

The goal of this study was to explore the potential role that technology can play in reducing the negative impact of social media on body image and ED. We conducted interviews with *n* = 12 experts by profession and focus groups with *n* = 18 experts with lived experience of ED. Through this paper, we provide unique insights into the perspectives of diverse stakeholders within the ED space, in the context of harmful social media content for people at-risk of or with ED. These insights are intended to inform effective content categorisation and the design of future technological solutions to mitigate the adverse effects of social media.

## Methods

We applied the concept of Information Power [51] to guide the decisions about participant sample size adequacy for this study. According to this concept, the higher the relevance of information that the participants hold to achieve the study’s aims, the fewer participants are required. Given the level of expertise amongst the participants and the quality of dialogue and depth of analysis, we considered the sample number enough to provide sufficient information power.

We first conducted interviews with *n* = 12 experts by profession. Through the interviews we narrowed down harmful content categories and their characteristics, challenges around the identification of this content and explored potential tools to mitigate the negative impact of social media. We then held a series of five focus groups with *n* = 18 individuals with lived experience of ED (experts by lived experience). Through these focus groups we further investigated their views on content categories relevant to ED originally identified by the experts by profession, and their perceptions regarding AI technologies as a potential tool for automating harmful content characterisation. AI was specifically narrowed in on for the focus groups as this was the main category of technology that the experts by profession identified in the interviews. For the experts by lived experience, we included all individuals who expressed interest in participating in the study to capture a broad range of perspectives.

### Ethics

We received ethical approvals from Monash University Human Research Ethics Committee (ID: 41131). All participants were provided with an explanatory statement about the study and asked to sign a written consent form. Before the beginning of each session, we additionally requested that participants provide verbal consent to audio recording.

To ensure the psychological safety of the lived experience participants in the focus groups, we imposed the following exclusion criteria for participation: (1) must not have an active ED, and (2) must be above 18 years old. We additionally sought in-depth feedback on all documentation and activity plans from our partner organisation, Butterfly Foundation (a national ED service provider in Australia), who reviewed the terminology and tone of language that we used and advised on the appropriateness of activities. They also provided us with necessary helpline services and instructions that we could provide the participants at the beginning of the focus group, to ensure adequate support provision should a participant become uncomfortable during the session. Finally, a Butterfly Foundation member attended the first focus group, as an observer, to ensure that our activities and the research team’s facilitation were adequate. Two research team members facilitated each focus group session. Facilitators had extensive experience conducting qualitative research within the themes of mental health, ED, and digital innovation.

### Recruitment

We recruited *n* = 12 leading body image and ED experts across Australia, by leveraging professional networks within the research team and a snowball sampling approach (i.e. the professional networks of existing participants). We emailed advertisements to gauge interest and interested parties were provided with the explanatory statement and an opportunity to ask questions to the research team. Interviews were scheduled at a convenient time for participants, with written consent obtained prior to each session. Participants received an AUD 20 shopping voucher as thanks.

For the focus groups, we recruited *n* = 18 individuals with lived experience of ED through Butterfly Foundation’s lived experience network, which encompasses people from Australia with a history of ED. Recruitment materials highlighted particular interest in speaking to traditionally underrepresented people in ED research (i.e. males, people from culturally and gender-diverse backgrounds). Interested participants filled out a Google form and were provided with the explanatory statement and an opportunity to ask questions to the research team. Focus groups were arranged at a convenient time for participants, with written consent obtained prior to each session. Participants received an AUD 60 as a token for appreciation for their time.

### Interview with experts by profession

We conducted the interviews via Zoom which lasted approximately one hour. It is to be noted that when we conducted the interviews, the Australian government had not yet passed the bill for banning social media for users under 16 years old [52]. The participants’ demographic details are provided in Table 1. The interviews focused on exploring four key areas: (1) Participants’ experience and the specific populations they work with; (2) Influence of social media on body image and ED; (3) Characteristics that might be useful for identifying social media content as harmful, ambiguous and safe; and (4) Potential solutions (digital or non-digital) to mitigate the negative effects of social media on body image and ED. The interview guide can be viewed in Supplementary Material 2.

Through a preliminary analysis of the interview data, supported by a review of existing literature, we generated eight content categories relevant to ED: (1) weight loss (including content influencing changes to body shape and size), (2) weight loss or performance-enhancing drugs, (3) ED recovery, (4) cosmetic surgery and other minimally invasive cosmetic procedures, (5) beauty tutorials, (6) food and nutrition, (7) exercise, and (8) body checking (i.e. where the creator is checking their body either in the camera, on weighing scales, or by measuring parts of their body).

**Table 1:** Demographics of experts by profession (interviews)

Code	Gender	Ethnicity or cultural background	Age	Lived experience of ED	Lived experience of body dissatisfaction
Int1	Female	White	25–34	No	No
Int2	Female	White	25–34	No	No
Int3	Female	White	25–34	No	No
Int4	Female	White	35–59	No	No
Int5	Female	Anglo/White	35–59	No	Yes
Int6	Female	Lebanese	25–34	No	No
Int7	Female	Bengali	25–34	No	Yes
Int8	Male	White Australian	25–34	Yes	Yes
Int9	Male	White	25–34	No	No
Int10	Gender-diverse (Non-binary)	Australian	25–34	No	No
Int11	Female	Australian	25–34	Yes	Yes
Int12	Female	Australian	35–59	No	No

Aligning with recommendations in [53], participants were asked to self-identify their ethnicity or cultural background and this was not standardised by the research team.

### Focus groups with experts by lived experience of ED

We conducted a series of five focus groups each lasting 1.5 h via Zoom, with *n* = 18 participants. We conducted focus groups 1, 2 and 3 with females (*n* = 3, *n* = 5 and *n* = 6, respectively), focus group 4 with gender-diverse participants (*n* = 2), and finally, focus group 5 with males (*n* = 2). We formed these groupings to foster a comfortable discussion environment. We explicitly asked participants from focus groups 4 and 5 if they would prefer to be placed in a mixed or gender-specific grouping and they chose gender-specific. We have provided the participants’ demographic details in Table 2. The focus groups aimed to examine the role of social media in developing ED and explore potential solutions to make social media safer. We used Miro boards (an online whiteboard that allows people to collaborate in real-time using diagrams, sticky notes and other tools to write down and organise ideas) to engage with the participants. The interviews with experts by profession fed into the planning of activities for the focus group.

**Fig. 1:**
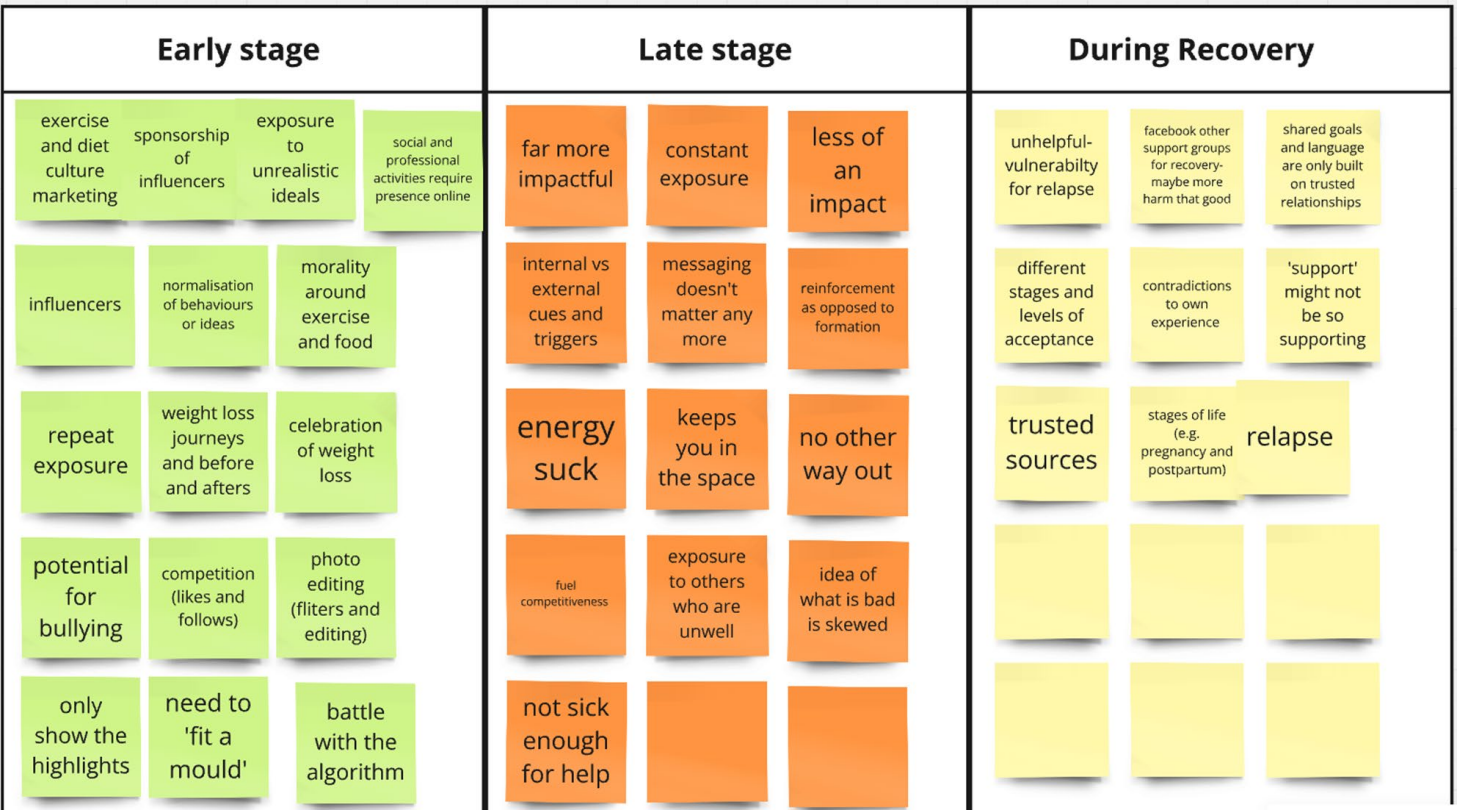
First activity of focus group focusing on the role of social media in different stages of eating disorder

For our first activity, we asked the participants to discuss the role that social media played at different stages of ED: early stage (i.e. where disordered eating patterns and challenging behaviours might be developing), late stage (i.e. where the ED is fully embedded and might require formal intervention), and during recovery. Through this we gained a crucial and nuanced understanding of how certain content impacts users differently depending on where they are in their eating disorder journey. The participants discussed both positive and negative aspects of social media for each of these stages, as shown in Fig. 1.

For our second activity, we asked participants to discuss as a group and create ‘We need’ statements for a hypothetical manifesto positioned to the government to advocate for safer social media platforms for those at-risk of or experiencing ED (inspired by methods in [54]). The participants then prioritised and selected their top three statements as a group. Through this activity, we aimed to understand what kind of harm was considered as a priority by experts by lived experience, and what they considered as safety and support, used to directly define design insights for moderation tools aligned with real-world experiences.

During our interviews with experts by profession, over half of participants provided ideas for the potential solutions to mitigate the negative effects of social media on body image and ED which revolved around the use of AI (e.g. use of chatbots to promote body positivity and provide automatic critical thinking tasks when certain images are detected in the social media feed). Thus, during the focus groups, we decided to explore the participants’ views on AI and the potential use of AI to create safer social media. Building on this, for our third activity, we asked participants to imagine that their manifesto (created in the second activity) was accepted by the government, who had then created an AI tool embedded in social media. This activity was intended to be a provocation to yield open discussion and prompt design insights. As such, there was no description provided about how the tool functioned. This loosely followed the values of invisible design approaches, an approach which is used to generate ideas and explore insights as a form of concept development [55]. We asked participants to consider AI use cases, how it made them feel, how it made their life easier, potential challenges, and prominent supporters and critics.

Finally, participants created rules for the AI to guide content moderation by identifying and categorising harmful social media content for the eight content categories, which we derived from the interviews with the experts by profession. They were asked to list ‘Must not show’ (prohibited content for social media) and ‘Can show’ (acceptable for social media) content for each category, helping us understand their views on what content is harmful or safe for social media. The focus group guide can be viewed in Supplementary Material 1.

**Table 2:** Demographics of the experts by lived experience of ED (focus group)

Code	Gender	Ethnicity or cultural background	Age	Religion	Type of eating disorder (ED)
FG1P1	Female	Australian	25–34	NA	Anorexia nervosa
FG1P2	Female	Australian/ New Zealand nationality of Scottish descent	25–34	Anglican	Bulimia nervosa
FG1P3	Female	European	25–34	NA	Anorexia nervosa
FG2P1	Female	Malaysian Chinese	25–34	Christian	Anorexia nervosa
FG2P2	Female	European Australian	18–24	Agnostic	Anorexia nervosa
FG2P3	Female	Anglo-Australian	18–24	Christian	Anorexia nervosa
FG2P4	Female	European	35–59	Catholic	Anorexia nervosa
FG2P5	Female	Indian	18–24	Atheist	Anorexia nervosa
FG3P1	Female	Caucasian	25–34	Atheist	Anorexia nervosa
FG3P2	Female	Australian	25–34	None	Anorexia nervosa and Binge eating disorder
FG3P3	Female	Australian	25–34	NA	Multiple types
FG3P4	Female	White British/ Australian	25–34	None	Other specified feeding or eating disorders (OFSED) and Orthorexia
FG3P5	Female	Caucasian Australian	25–34	NA	Anorexia nervosa
FG3P6	Female	Iraqi Muslim	25–34	Islam - Shia sect	Anorexia nervosa + Avoidant/restrictive food intake disorder (AFRID)
FG4P1	Gender-diverse (Transgender man)	Australian with a Greek cultural background	25–34	None	Anorexia nervosa
FG4P2	Gender-diverse (Non-binary)	Maori, European	25–34	None	Bulimia nervosa
FG5P1	Male	Sri Lankan, Australian	25–34	Buddhist	Anorexia nervosa
FG5P2	Male	Caucasian	35–59	None	Anorexia nervosa

Aligning with recommendations in [53], participants were asked to self-identify their ethnicity or cultural background and this was not standardised by the research team.

### Data analysis

Across the study, we had 19.5 h of audio-recorded data. The first author transcribed all the audio recordings and de-identified the transcripts. After the completion of the interviews with experts by profession, the first author used the Framework Method [56] guided by inductive thematic analysis [57]. Initially, the first author familiarised themselves with the data from the experts by profession and developed preliminary codes at the paragraph-to-sentence level using NVIVO software. The remaining research team also familiarised themselves with the data and collaboratively reviewed the initial codes. The Framework method was then applied to systematically manage and interpret the data. This involved developing a coding framework, charting data into a matrix, and identifying patterns and relationships across participants to enable structured comparison and synthesis. After iterative coding, mapping, scoping of the relevant literature and numerous discussions a set of content categories was generated. These categories were further explored in the focus groups with experts by lived experience. After the completion of the focus groups, the same Framework Method was applied. The first author used inductive coding to identify and organise key themes within a structured analytical framework. The research team collaboratively reviewed, discussed and refined the resulting framework and theme until a consensus was reached on the final significant themes.

Data from the existing literature, interviews and focus groups were triangulated and are presented as such in the findings [58]. The interviews provided in-depth academic and professional knowledge and reasoning behind harmful content categorisation, as well as insights around opportunities for technology to mitigate the negative effects of social media. The focus groups then added a crucial layer of nuance by providing personal perspectives from those with lived experience of ED. The triangulation confirmed the developed themes and increased the credibility of the findings [59].

### Researcher reflexivity statement

As authors, we acknowledge that our identities, experiences and lived experience with body image have shaped how we approached this study and interpreted the data. Our professional engagement with digital health and mental health research influenced how we understood the role of technology in mitigating the harms associated with eating disorders. We recognise that our interpretations were co-constructed through the combination of our perspectives and the participants’ expertise and experiences, rather than existing independently of them. Through iterative reflexive dialogue, we critically examined the influence of our perspectives, disciplinary backgrounds and values around technology and wellbeing, and challenged our assumptions. Rather than seeking to eliminate the subjectivity, we viewed reflexivity as a resource that deepened our understanding and strengthened the credibility and contextual sensitivity of our interpretations.

## Results

In this section, we consolidate the findings from interviews and focus groups. For easy identification, we have added prefixes to the participants of interviews and focus groups: interviews (Int) and focus group (FG). Throughout our findings, we refer to participants from interviews as experts by profession and focus groups as experts by lived experience, to further add clarity regarding where data was derived. Six core themes from across the data are reported below: (1) Spectrum of harmful and ambiguous content on social media, (2) The “echo chamber” of harmful content amplified by social media algorithms, (3) Balancing safety, freedom and responsibility in social media moderation, (4) Shared responsibility and collaboration for safer social media environments, (5) The role of representation and diversity in social media recovery and support, and (6) Harnessing digital innovation to reduce harm on social media.

### Spectrum of harmful and ambiguous content on social media

The experts by profession stressed that understanding social media content can be nuanced and subjective, which becomes a challenge in differentiating between harmful and safe content; *“the fact that it’s not black and white. […] People might have different perspectives on whether something says red [harmful] or amber [ambiguous] or even green [safe]”* (Int12) and *“I guess this is the problem because it wouldn’t be harmful for everybody […] So*,* there would be a lot of people that look at that content and would be absolutely fine and not harmed at all.”* (Int2). Int2 suggested that to overcome this challenge, the potential solution could be to separate harmful content categorisation for different population groups: *“whether we categorise as harmful for everyone or harmful for people ‘at-risk’. So*,* for example*,* people with ED*,* people at-risk of ED*,* or people in recovery could be part of that kind of ‘at-risk’ group*,* and then you have kind of a general one”* (Int2).

Participants broadly discussed a range of different social media content types, from generalised content that might be considered normal viewing on social media platforms (e.g. healthy eating recipes that provide nutritional guidance, exercise videos, and beauty tutorials) to more obviously challenging content (e.g. the promotion of weight loss drugs and cosmetic procedures, very underweight creators performing body checking behaviours - weighing, measuring, pinching parts of their body, or generally looking for signs of visible fat). Concerning more generalised content, which could be considered ambiguous in its impact, the experts by profession agreed that the intention of the video was vital:

*“They [fitness influencers] are more ambiguous because while people may take that as inspiration and use that for a healthier lifestyle to get more in shape and to have just a better physical health*,* it can be negative for those who are obsessed with exercise and engage in excessive exercise that is not necessary for them. And a lot of that exercise is driven by appearance more so than driven by health”* (Int7).

The experts by profession suggested that ambiguous content starts moving towards harmful when it starts focusing towards comparisons like ‘before and after’: *“we find that [before and after] really toxic obviously because they’re saying that in the first image they’re not worthy. They’re not worthy unless they lose weight and head towards that after image”* (Int11) and content promoting very low-calories diets: *“Some content might have meal plans that follow really low-calories meal plan […] if people are encouraged to follow that plan to lose weight or because they’re not worthy of their current appearance*,* that can be really damaging”* (Int11). In addition, accompanying textual representations of elements such as the creator’s weight or body measurements, the calories content of foods, and calories burned, were often seen to be the potentially harmful element, even if the overall tone of the video was more positive: *“Anything that shows numbers like if someone talk about how many kilograms they weigh and what their goal weights are*,* or showing how many calories they might be eating*,* or how many grams of sugar”* (Int5) and *“I think stuff using quantitative numbers*,* things like that should probably be banned. Anything where someone says their weight or calories limits should be banned”* (FG2P3). The experts by lived experience further clarified why showing numbers in such content can be so damaging: *“As somebody who weighed their food and counting calories for years*,* it took forever to get out of that[…] a lot of the times these people are guesstimating calories. It just doesn’t need to be there”* (FG2P5). Perhaps the most extreme form of content was that depicting body-checking behaviours from creators who would be considered underweight: *“encouraging people to take their headphone cord and see if they could wrap it around to their stomach and if they were able to reach the full head headphone cord that was good*,* they were skinny”* (Int11).

‘What I eat in a day’ videos (a common trend on social media depicting videos around what a person eats throughout the day) were seen as particularly challenging across the entire cohort of participants: *“because everyone’s different. What might be sufficient for me to eat in one day may not be sufficient for someone else to eat in one day. It doesn’t take anyone’s history or medical conditions or cultural aspects in consideration”* (FG3P6). Participants stressed that any type of diet plan or health advice should come from experts with proper credentials, or have a disclaimer that it is not applicable for all:

*“even if someone is qualified to be giving that advice*,* they don’t know who’s going to be taking it in”* (Int11); and *“a lot of these health gurus that might not even be trained in certain areas about food or nutrition and like targeting a lot of vulnerable people to buy certain products which can also lead to body image concerns”* (FG4P1).

The experts by profession additionally highlighted several other significant body-related trends, such as the ‘A4 Waist Challenge’: *“A trend that involved getting an A4 piece of paper and holding that up in front of your body as well. And if your body was larger than that vertical*,* a four piece of paper*,* you would be shamed. If it was smaller*,* you would obviously be praised and considered that’s an ideal”* (Int11) and the promotion of thigh gaps as a body ideal: *“Thigh gaps*,* which I think now has kind of transitioned into legging legs. […] do you have like a big thigh gap*,* and they’re just like straight twigs?”* (Int1).

Content promoting weight loss drugs (like Ozempic and Mounjaro), or performance-enhancing steroids, to aid in increasing muscle mass, were seen to be extremely harmful and often facilitated the spread of misleading information, that could put one’s health at risk:

*“If someone*,* for example*,* is saying “Oh!! take Turkesterone [help increase muscle mass and strength]*,* it’ll boost your testosterone*,* you’ll get mad gains”*,* there’s zero evidence for the safety and efficacy of that. Therefore*,* that is misinformation*,* and that kind of content is harmful either on misleading ground or because it’s intensely negative”* (Int9).

The experts by lived experience were vehemently against showcasing any advertisements related to weight loss drugs: *“It will put them [any viewers] at risk […] I don’t know why an advertisement would be needed”* (FG3P2) and *“I would love to see that just not exist on social media personally”* (FG4P2).

When it came to content discussing ED recovery, there were mixed views. Participants made it clear that ED recovery content showcasing medical components or hospital settings should be considered harmful, as it provides a source for comparison of symptoms. If not presented carefully, this could be considered triggering, or indeed a motivator for people at the earlier stages of their ED: *“Any content that is based in a hospital setting or implies that someone has to go to a hospital for their ED to be taken seriously*,* that would obviously be toxic”* (Int11) and *“They’re doing XYZ*,* so this means that they have an ED*,* whereas I don’t have XYZ symptoms or I don’t have those food rules. So*,* therefore maybe I don’t have an ED. I’m just spiralling for no reason and I don’t actually have to get help*” (FG2P5).

Finally, a surprising source of ambiguous content that was discussed was beauty tutorials. While beauty tutorials don’t directly impact ED, they can cause body dissatisfaction and strengthen the ‘idealised’ concept that can cause serious harm to society:

*“We’ve seen an influx of 12-years-old going into Mecca and Sephora and wanting to buy retinol [anti-ageing product] and ingredients that are just way beyond their age. […] We often think about body image as being something from the neck down. But*,* I think social media has tried to include everything”* (Int4) and *“Definitely harmful*,* obviously for emphasising that appearance is super important*” (FG3P3).

This broader preoccupation with appearance was seen to drive some individuals further along the spectrum toward more clearly harmful behaviours to attain an ‘ideal’ that could potentially lead to more drastic steps, such as unnecessary cosmetic procedures: *“content that encourages dieting or*,* things like unnecessary cosmetic or medical procedures to appear a particular way”* (Int4).

### The “echo chamber” of harmful content amplified by social media algorithms

An ED “echo chamber” refers to a digital space where content normalising and promoting disordered eating behaviours are continuously shared, such that the individuals in the space are repeatedly exposed to content which further reinforces their disordered eating beliefs and behaviours. Participants in all phases discussed the development of an echo chamber of sorts, created through continuous viewing of certain content types, which served to normalise unrealistic body standards and feed into existing belief systems: *“it’s going to create these appearance norms where people think ‘Ohh!! Everyone looks like this and I should look like this’. So it’s adding to these pressures*,* both in terms of the body size and facial appearance”* (Int1) and *“The more we’re exposed to them [unrealistic ideals]*,* I think the more they’re perceived as normal”* (FG1P1). The experts by lived experience brought a new perspective around how certain types of content could create a sense of competition in individuals at-risk of or with ED: *“There is that culture on social media of competition to see who can be the skinniest*,* who can recover the fastest*,* or who can do whatever. It’s a real competition to keep up with those things”* (FG2P4).

The echo chamber of ED content starts to blur the boundary between reality and harmful ideals, with filters and photo-shopped content playing a leading role in perpetuating unrealistic standards: *“As a society as a whole*,* the less realistic*,* the less attainable*,* the more photo-shopped*,* the more filtered tends to be unhelpful because it’s portraying something that’s not actually real or achievable for the vast majority of people”* (Int12). It distorts reality to the point that, when individuals come face-to-face with reality, they cannot accept it:

*“I got at a certain point*,* I kind of got used to the filtered version of me. So when I looked in the mirror and saw the real version*,* it was quite upsetting for me and also that sense of discomfort of not being able to post an unfiltered picture when you get used to the filtered version of yourself”* (FG4P2).

The disconnect between filtered social media content and how individuals appear can lead to people feeling as though they don’t measure up: *“with filters to hide cellulite or wrinkles or whatever it might be. I think for those vulnerable people continually seeing these types of images makes them feel as though they’re sort of outside of the box if they don’t fit that mould”* (FG1P3).

The inherent harm of social media algorithms within this context further highlighted this point. If viewers are watching certain types of harmful content, algorithms are learning this pattern and consequently exposing them to similar content, creating a feedback loop that intensifies harmful narratives: *“It’s not just the content*,* it’s the algorithms that recommend and deliver content and the ways that people engage with that content*,* whether consciously or unconsciously*,* that makes it more likely that the content will be delivered to them”* (Int9). Existing biases in the algorithm itself can also serve to further strengthen the likelihood of harmful content reaching certain demographics:

*“The algorithm is gender biased*,* so it will show young girls who have not searched for anything or followed anything in relation to diet and nutrition*,* it will show girls diet content very early on in their journey on the platform*” (Int5) and *“there have been some suggestions that there is some dark stuff happening around censoring non-white creatives”* (Int1).

Trying to combat this echo chamber, created by the algorithm, was something the experts by lived experience found significantly challenging, particularly in the recovery process. The continuous bombardment of such content threatened, and potentially penetrated, the shield they had formed against these concepts:

*“There is this whole thing of constant everyday battle with your algorithm. As soon as you open anything […] and no matter what you’re doing*,* you’re still opening up feeds that are going to have things in it [harmful]*,* so you’re still put in that space where you’re having to combat and filter and challenge all of these messages. So*,* there’s still a big drain that comes from it”* (FG1P1).

For some, this led to a withdrawal from social media altogether in an attempt to protect their recovery.

As discussed by the experts by profession, comments left by other social media viewers on creators’ content were often seen to be as harmful as the content itself: *“I find with a lot of toxic social media content*,* especially on TikTok*,* you’ll often get comments as well that are just as toxic*,* if not more toxic than the actual video.”* (Int11). These comments can amplify negative views surrounding body image, acting somewhat as evidence that a person is not good enough in the eyes of society: *“the comments that come through is a very clear indicator of the public perception or opinion on whatever this main topic is”* (Int6) and *“you’re seeing someone else getting negative comments who doesn’t make those ideals and that can make you feel negative*,* even if those comments are not directed at you specifically”* (Int10). Even when the content itself was seen to have positive components, such as promoting encouragement to live a healthier lifestyle, the negative comments were seen to overpower the positivity of the content itself. This phenomenon was particularly notable in content made by creators living in larger bodies: *“The post that generates those kinds of conversation can be a positive post like it could be a fat person enjoying life or a fat person getting married or succeeding*,* but there will still be comments underneath that are fat phobic*,* negative and abusive towards that person”* (FG4P2).

### Balancing safety, freedom and responsibility in social media moderation

The experts by profession expressed frustration over their experiences with content reporting, with the manual scouring and flagging of content a resource-intensive process:

*“And so they [social media platforms] come and say we need more information*,* that is really time and resource heavy. It takes a long time to then go back through especially if you need to go back through lots of different videos and comments. […] Certainly*,* the time and manual aspect of it would be a challenge”* (Int11).

It was often the case that social media platforms refused to remove harmful content, citing their platform guidelines as a justification rather than trusting the expert opinions of ED specialists:

*“Someone in our community found this person on TikTok who was doing these years of body checking*,* and this person was really*,* really underweight […] quite a lot of the comments were full with people saying ‘give me your diet tips’ - that sort of thing. And her account was just dedicated towards these body checks. However*,* she didn’t mention eating disorders in the caption and she didn’t talk about eating disorders at all. It was only the people commenting that mentioned eating disorders. Since she didn’t mention eating disorders*,* TikTok said that it did not go against their guidelines”* (Int11).

Of course, in many cases, particularly with more ambiguous content, creators are not aware that their content could be harmful to people experiencing or at-risk of ED. However, in others, creators deliberately try to evade social media safeguards to keep their harmful content online, through methods such as mutation or avoidance of hashtags: *“People mutate those hashtags. So ‘#anorexia’ might now be displayed as [an0r3xia]*,* but the ‘O’ is a zero or the the ‘E’ is three”* (Int11) and *“A lot of people on Twitter will post what is ostensible ‘thinspiration’ and not hashtag it with particular things because they know that if people see it*,* who might not have an active eating disorder or be actively kind of restricting*,* they’ll report it and they’ll get removed.”* (Int8). Even banning certain harmful hashtags does not guarantee proper blockage of harmful content. The experts by lived experience drew this concept out further, sharing incidents of ‘hashtag hijacking’:

*“I think it can be difficult when hashtags that are meant to be helpful*,* especially for hashtags saying recovery for eating disorders are hijacked by influencers or people promoting things like weight loss. […] Concepts of ‘intuitive eating’ is like ‘intuitive fasting’ now and that’s really unhelpful”* (FG3P4).

There is a distinct tension surrounding social media platforms in regards to maintaining a safe online environment for viewers and not implicating freedom of speech:

*“I think the freedom of speech is definitely a big one and definitely something that I’ve felt from the respective apps [social media platforms]. That is a very big concern of theirs and they want to keep that in check so they’re not censoring their users to such an extent where they will go to a different app to share their story and talk about the same things”* (Int11).

This freedom of speech differs from country to country, which might pose a challenge for content moderation more broadly:

*“There’s a cultural element to that as well*,* and that a lot of these companies are American-based where the cultural component of freedom of speech is so strong and powerful. I think in Australia*,* we’re more used to being censored*,* and we’re more comfortable with the benefit that can have for protecting vulnerable people. But I think in America the swing is towards that freedom of speech element”* (Int12).

However, the experts by lived experience were adamant that ‘freedom of speech’ should not be an excuse provided by content creators and platforms to push harmful content, that could ultimately affect someone’s life:

*“All the pushbacks towards governments trying to introduce greater punishments and regulation for these platforms in the name of freedom of speech*,* but the big argument is if it impacts people’s mental health badly then you can’t cause millions of people [harm] by affecting their mental health and get away with it completely. There needs to be some sort of accountability for both the creators and platforms”* (FG2P3).

Building on this, participants also felt that the corporations and content creators who are beneficiaries of diet culture and societal beauty standards, and who rely on advertisements and content reach to promote products could potentially be the barrier for resistance to content moderation:

*“I think a lot of people would have difficulty with being told that they can’t post certain things or seek out certain information. [.] I think the diet culture is so prevalent that fills the pockets of industries. Individuals will struggle to adhere to the rules”* (FG3P1) and *“People*,* organisations and companies whose income and profit is based on promoting harmful content on social media […] I think would be quite a critic here”* (FG5P1).

The experts by lived experience stressed that much of this harmful content is exploitative in nature, designed to take advantage of people’s emotional vulnerabilities for profit: *“it’s driven by exploiting someone’s emotional state. It’s all hype and bubble […] it shouldn’t be in the hands of someone who’s making profit”* (FG5P2).

### Shared responsibility and collaboration for safer social media environments

Participants in all phases mentioned the need to bring government and social media platforms into the agenda to make social media space safer. The needs ranged from making the government aware of the situation *“can we go to policymakers and say that this type of content is either safe or ambiguous or might help to mitigate some of the onslaught of idealised content that’s pushed to these demographics”* (Int4) and making it compulsory for platforms to open their API (Application programming interface) for research *“By giving us access to things like the API and*,* within reason*,* how the algorithms work*,* research can be more specific… higher quality; and policies that are implementable and useful and that don’t put undue burden on platforms can be brought into being”* (Int9). Additionally, Int9 also discussed how researchers could aid the government in bringing platforms into the loop, by providing them with solid evidence:

*“The more specific we make our research and the better we can articulate mechanisms*,* the easier it is for policymakers to move forward*,* because if we present them with findings that are tantamount to*,* ‘social media*,* on average is weakly positively correlated with body dissatisfaction’ - What can they do with it? They can’t ban social media. They don’t know what to even tell the social media companies. So that’s the best thing we can do is to push ourselves for methodological complexity and specificity in the research”* (Int9).

All participants agreed that there was value to social media, and future interventions needed to work with, rather than against, the benefits it could provide: *“They’re [young people] born in the age of social media. We just need to work out”* (FG2P4) and *“because then you miss out on the positives that social media provides as well. […] you lack that connection with certain people and stuff like that”* (FG3P1). The experts by profession, in particular, highlighted how responsible content creators were useful vehicles for promoting positive behaviours: *“They [responsible fitness content creators] do have ideal bodies themselves […] but they are doing it in the most healthful way that can be done. And what I like about that content is that it’s practical and has a really significant reach”* (Int9).

The experts by profession also emphasised the necessity of collaborating with content creators to promote responsible content generation: *“I think that would be really great if we could see a culture shift amongst influencers and content creators as to what they’re posting*,* how they’re posting it and if it’s done in a safe way”* (Int11). However, the experts by lived experience described that current safety measures that content creators often provide were not enough: *“I agree with filtering out things before they get to being in front of the person cause things like trigger warnings and blurred posts don’t do anything”* (FG3P1).

### The role of representation and diversity in social media recovery and support

Participants in all the phases stressed that it was necessary to see more diversity in social media content, in terms of body shape, size, colour and gender: *“I think it’s healthy to see diversity of body shape and size”* (Int12). Diversity is essential to normalise realistic body standards and create a sense of belonging and connection for the viewers. This sense of belonging is particularly significant for ED recovery:

*“I didn’t see a single person of colour talking about their ED. And I think that just definitely felt like I was alienated even more”* (FG2P5) and *“I wasn’t able to see myself represented anywhere in stories of hope. So*,* then I didn’t think there was hope when I was in recovery”* (FG4P1).

This concept of diversity needs to be more broadly expanded towards underrepresented populations in the ED space (culturally diverse, males, gender-diverse and differently-abled people):

*“it was females who were like White and upper class and that they were the people that I’d see share their stories*,* but I never really saw any diversity of people with disabilities or men or people with different gender and sexuality or different race or homeless or anything like that”* (FG4P1).

The experts by lived experience also shared how not being represented in promotional materials for support services on social media could create a barrier for seeking help: *“A lot of body neutrality posts are geared towards young women. I think a lot of support services are geared towards young women as well […] I held a lot of shame around accessing services because I was a guy who was experiencing something like that”* (FG5P1).

The lack of representation becomes further complicated when intersectionality comes into play:

*“When you’re a person who experiences multiple types of disadvantages. So*,* for me*,* it was my body size*,* you know*,* being a fat person*,* but other things like my ethnicity*,* my facial appearance my body shape*,* socioeconomic and all other things*,* I think it almost placed a greater pressure on me to try and be beautiful because I can’t change these others things about myself […] it felt like the only the only way I would ever be able to join the realm of the acceptableness”* (FG4P2).

### Harnessing digital innovation to reduce harm on social media

When asked about the technologies that might aid in mitigating negative impacts of social media, participants discussed the value of automation to support the time-intensive process of moderation: *“AI could go in and kind of just flag anything that could potentially harmful*,* so that as a moderator*,* you’re not having to constantly go through because it’s time consuming”* (Int4) and *“If it [AI] could identify that kind of content [harmful] and remove it completely”* (FG2P1). The experts by lived experience additionally suggested an option for filter removal: *“remove like filters that alter your body and appearance so then everyone comes through as who they actually are”* (FG4P1).

The experts by profession also valued the potential screening and tracking capabilities of technology, for automating the detection of viewers’ behaviours: *“detecting that there’s an issue for someone by looking at their patterns of usage of online social media”* (Int6) and identifying patterns of viewing harmful content: *“If you can see this*,* you can present this to the user and you can try to deliver notifications that move people somewhere else”* (Int9). The experts by lived experience had similar thoughts: *“screen if someone is searching for particular content [harmful] and maybe alert a psychologist that they might need support or follow-up”* (FG2P2).

For the experts by lived experience, having a level of personalisation was vital to support individual needs: *“like having some personalisation so that it can be customisable because it’s very different for everyone”* (FG4P2), suggesting a form of of feedback system to support better recommender systems: *“Maybe if afterwards or during [watching content]*,* you’ve got the option of AI asking*,* ‘Is there something you didn’t like in this video? That you want us to hide in the future’.”* (FG1P3). In their view, using technology with added safeguards would give them back control and make them feel safer:

*“I think it would make me feel more willing to spend time online and engaging in online spaces. There’s so much stuff that I don’t have control over*,* and it’s not like I don’t know.”* (FG1P2); *“Probably feel more at peace*,* like less consumed by like all this misinformation and be able to trust myself more”* (FG4P1); and *“Well*,* mine would probably be AI that alters the algorithm to show people content that uplifts them”* (FG4P2).

Participants also saw this as a chance to use algorithmic manipulation to populate social media feeds with positive and happy content: *“you limit to only the stuff that brings you joy*,* the stuff that captures your attention or keeps you watching that brings you joy and we’re just going to show you that”* (Int1); and *“Anyone who’s coming up with this thing [AI solution]*,* would have spent enough time to work out the plotted chart of where it leads to. So*,* if that information is available to people to know where it’s going and what is the intention behind it”* (FG5P2).

However, the experts by profession had some concerns regarding the technology itself in terms of its performance, due to the subjectivity of what might be considered harmful: *“I worry that the machine learning wouldn’t be able to distinguish between stuff that was harmful and not harmful because there’s such a nuance to that. I think probably as I’ve been talking and it’s very hard to quantify what is actually harmful or not”* (Int10). They emphasised the need for human intervention in specific scenarios, rather than relying solely on the technology to take specific actions: *“I guess ideally if someone is expressing very troubling things to a chatbot [technology]*,* it should sort of connect them with someone that they can speak to*,* like a real person”* (Int3). Int3 further added that technology has to be appropriately evaluated and validated before use: *“As long as there was lots of piloting of it*,* then I think I would feel OK”* (Int10). There were also some concerns from the experts by lived experience regarding using AI in clinical settings:

*“I have concerns around how AI is used in diagnosis and treatment. But*,* I think I would perhaps look at AI being like the prevention side of things and identify the systems that currently exist that are creating harmful environments for people around their body image and ED*,* or maybe at the risk of ED”* (FG5P1).

## Discussion

Our study aimed to explore the different contextual factors within harmful social media content for people at-risk of or experiencing ED, inform content moderation strategies and assess the potential role of technology in reducing the negative impact of social media. Our discussion is structured as follows. First, we discuss how there is a need for shared responsibility between different key stakeholders and how technology can support that shared responsibility in content moderation. We then describe opportunities for integrating diversity and culture into social media, to create a more inclusive and diverse environment. Finally, we explore how the use of understanding these nuanced contexts could support novel approaches towards the design of future interventions to protect social media users experiencing, or at-risk, of eating disorders from harm.

### Principal findings

#### Need for shared responsibility in content moderation

Our findings indicate that filtering harmful content should not fall solely on individuals with ED or their supporters. Instead users, creators, platforms, and government all share responsibility for creating safer online environments. While users can engage in algorithmic manipulation, for example, by curating their feeds through ‘digital pruning’ [42], unfollowing, muting or blocking harmful accounts, and deliberately following content or creators that don’t focus on appearance (e.g. instead focusing on content that aligns with one’s hobbies and interests) [60], these strategies alone are insufficient. As our participants stressed, focusing on only the users to curate their content and think about harmful content in a critical way is not enough, as this runs the risk of exposing potentially vulnerable users to harmful content in the first place, which is a known risk factor for ED formation and exacerbation [61, 62].

To ease the burden on the individual, we could potentially use automated technology such as mobile sensing approaches and automated monitoring of social media behaviours (e.g. Facebook’s suicide prevention algorithm which browses through user posts to detect potential suicide risks [63, 64]). Besides this, automatic prompts for wellbeing and resources could be potentially triggered when a pattern for repeated exposure to harmful content is detected. The experts by lived experience in our study mentioned that they were not apprehensive to use automated social media monitoring if it meant that they would be safe from harmful content. Similarly, Vega et al. [65] reported that their participants with binge eating and bulimia showed high levels of acceptance for automated sensing approaches and engagement with reflective activities and contextual logging. The findings of this study align with our results, highlighting the potential of context-aware systems in monitoring and reflecting on social media behaviours which could be a promising area for further research.

Policy also plays a critical role. The recent Australian ban on social media for children below 16 years [52] reflects the increasing governmental awareness of online harms and emphasises a responsibility for social media platforms to actively engage in safeguarding vulnerable populations from online harms. But aside from from the age-based protections, it is crucial to address the needs of other vulnerable groups such as individuals who are at-risk of or experiencing ED and who may fall outside the ‘under 16’ age bracket. Ensuring online safety for all users requires a collaborative approach, where both government policy and platform accountability come together to address the online harm.

As our findings showed, the reality is that despite features like unfollowing, muting and blocking in platforms, loopholes in platform guidelines still allow some creators to bypass moderation systems. These bypasses can lead to viewers being recommended similar content again [66]. As a solution, platforms could provide a ‘reset’ algorithm option, which would help the algorithm unlearn patterns and behaviours of the viewers and start afresh. For example, if an individual is diagnosed with ED or finds themselves in a period of ED relapse, then they can reset their social media algorithm rather than cutting off social media altogether. As expressed by our lived experience participants, this could be especially valuable during the recovery stage of ED, when users are particularly vulnerable to the impacts of harmful content.

One factor that future researchers conducting content moderation work need to be mindful of is that moderation efforts often face criticism under the banner of ‘freedom of speech’. There is an ongoing battle between ‘freedom of speech’ and public safety and well-being. Kozyreva et al. [67] explored the critical factors that can tip the scales between these conflicting interests: the extent of harm, frequency and repetition of conducting harm, and the content category. Creating blanket bans on content categories is highly infeasible as without proper critical thinking skills regarding its harm, users will figure out ways to circumvent blanket bans [49]. Such blanket bans could potentially censor recovery content that could be highly motivating and useful for individuals at-risk of or experiencing ED [68]. Instead, future research could focus on supporting users and moderators (like our experts by profession) to move towards automated data collection rather than self-reporting, so that they can present solid cases to social media platforms for required content bans. The automated data collection may prove useful for generating reports that drive government support for restrictions on certain social media content. For example, in a report from ‘Reset Australia’ [69], the authors presented an experimental study where paid-for-advertisement approval systems from various platforms approved their pro-ED advertisement. Building technologies that support evidence collection (e.g. tracking and analysing approval processes for ads, interrogating and analysing platform guidelines and extracting comments from social media posts) may be crucial for driving accountability and regulatory action.

#### Enhancing diversity to break out of the echo chamber

Our findings showcased the importance of bringing diversity (in terms of body shape and size, culture, gender, and ability status) into social media feeds to help support an escape from the echo chambers that social media algorithms create. Enhancing diversity not only serves to blur the lines between what is considered ‘ideal’ and the reality of how society is made up, but also helps people who might be outside of the stereotypical white, female and anorexic vision of an ED [70] feel connected during ED recovery. However, like many AI-driven algorithms, social media algorithms are inherently biased. The findings from our research resonate with multiple research studies, which have found social media algorithms to be biased towards creators with disabilities [71], larger bodies [72], from different racial backgrounds [73] and from LGBTQ + communities [74]. This bias, which sidelines creators from diverse backgrounds, can limit viewers’ exposure to content that might reflect their own identities (e.g. gender, sexuality, physical appearance, body functionality and cultural background). Thus there is a need to provide access to marginalised content creators in order to normalise variance and avoid confinement towards restrictive beauty standards. Recent work by Shrestha et al. [75], which co-designed digital interventions for body image with underrepresented populations, highlighted the vital importance that exposing individuals to diverse bodies can have in promoting positive body image. Their participants showed high enthusiasm for future interventions that supported increased diversity in social media (in terms of representation of diverse creators and non-appearance-related content).

Biases can also be addressed through using diverse datasets during model training to minimise gender and cultural biases [76]. AI developers, in particular, should provide specific considerations to ensure their training data has fair and accurate representations of diversity. Cramer et al. [77] present helpful checklists for AI developers to help them think critically about diversity and representation by considering the purpose of the system, the output of the system and its impact on society and users before they begin developing it. However, a significant challenge in addressing these issues lies in the lack of algorithmic transparency on social media platforms. The opaque nature of these algorithms creates challenges for researchers and policymakers to assess what data is being used in training and how it influences content curation and recommendations [78, 79]. This hinders efforts to identify and correct the biases. Despite this, it remains essential for developers and platforms to be mindful of these concerns and proactively adopt inclusive practices such as sociotechnical transparency frameworks [78] to create socially responsible AI systems.

Recent work by Soubutts et al. [53] on digital mental health service design with culturally diverse young people presented a call to action for researchers to actively consider the engagement of culturally diverse people in technology design. During technological development, it is important to ensure that solutions resonate with individuals’ needs, requirements and experiences. This is even more important in body image and ED, with significant gender and cultural biases in its literature base [75, 80].

#### Understanding the context of harmful content

Our findings indicated there is a need to create a robust set of rules for what is considered harmful and safe for people at-risk or with ED. This classification can be utilised to train algorithms to perform better content moderation, moving the field away from a reliance on hashtags and keywords. Evading hashtag moderation by using mutated hashtags, avoiding hashtags altogether, or hijacking and using hashtags meant for different content (e.g. body positive social media content) has been a go-to method for specific content creators and advertisers to keep their harmful content online [66]. However, before we can delve fully into the classification of these content, it is essential to understand the context surrounding certain content to ascertain whether it is harmful or not.

Existing algorithmic moderation systems overwhelmingly focus on identifying the most explicit forms of ED-related content (e.g. pro-ana, thinspiration), leaving subtle, ambiguous or context-dependent material largely undetected. Our findings highlight that the majority of harmful content that our experts encountered or discussed sits outside these extremes. This content can reinforce disordered eating behaviours not because of what is shown, but because of how it is framed or interpreted by vulnerable viewers. The insights generated from this study point towards the need for future algorithmic approaches that are capable of recognising contextual signals and patterns of exposure over time. Rather than relying solely on surface level like hashtags or highly explicit visuals, more sophisticated and context-sensitive scanning approaches are needed to capture nuanced content.

Prior research has explored the use of AI technologies to generate captions and descriptions for image and video content by understanding and exploring the content’s context [81, 82]. This research can be leveraged in future work surrounding image-based social media classification by bolstering this generic contextual understanding with rules for classifying harmful content. This way the AI will move further beyond keywords and hashtags to include the intent and tone of the content. For example, model alignment (a process of embedding human values into AI models and manipulating its output to align with human goals and rules) [83] within generative AI systems that can provide textual descriptions of videos and images (e.g. videoLlama, chatGPT). The implementation of highly robust methodologies used in healthcare research, such as the Delphi technique [84], which is a systematic process for gathering consensus, could be used in future research to drive the development of rules that are highly regarded as relevant and necessary for future AI systems aiming to classify harmful content.

We propose that a useful direction for future research would be to develop AI systems that could be used by content creators. Rather than focusing solely on moderating harmful content after it appears on social media, it would be more effective and practical to prevent such content from being created and uploaded in the first place. In line with this preventative strategy, the Butterfly Foundation, in partnership with Instagram, has launched a social media series featuring Australian content creators who share their experiences with mindful content creation to avoid unintentional impact on body image perceptions, with a focus on promoting the well-being of their audiences [85]. This highlights the importance of educating content creators to be more aware and mindful of how their content can influence viewers, fostering a more responsible and supportive digital environment.

## Limitations

​​Despite recruitment efforts to recruit participants from diverse backgrounds for expert interviews and focus groups with individuals with lived experience, most of our participants were White and female. In addition, the majority of our lived experience participants had experiences of anorexia, meaning that the experiences of participants with bulimia, binge eating disorder and other ED classifications were not fully represented in our sample. While our research had a diversity level in its sample, our numbers were small. While a challenging task, it is vital that future research attempts to amplify the voices of these traditionally underrepresented voices in ED research. Approaching multiple lived experience networks, or recruiting directly from formal ED services, particularly those that focus on more marginalised experiences of ED, might aid more diversity in recruitment samples in the future. We did not collect data on participants’ sexual orientation in this study. However, this could be considered in future research to provide a more comprehensive understanding.

## Conclusions

Our paper reports on the range of contexts of potentially harmful content in social media for individuals at-risk of, or experiencing ED. The understanding of these contexts aims to empirically inform the future classification efforts of such content, and the role of technology in supporting this process. We have presented unique insights into the nuances and subjectivity of such content, and the factors that could tip the balance between harmful, ambiguous and safe content. A key contribution of this paper is its explicit focus on the ambiguous or “grey areas” of social media content which are very subtle and context-dependent that the current algorithmic systems usually overlook them. It provides a foundation for developing more context-aware, nuanced and user-sensitvie approaches to content moderation. This paper also presents potential technological solutions for future research to mitigate harmful social media content. However, challenges remain in developing technological solutions that safeguard against harmful content, while maintaining balance with ‘freedom of speech’ and avoiding censorship. We also emphasise the need to enhance the diverse representations in social media, by focusing on the promotion of non-appearance-related and diversified content regarding body shape, size, gender, body functionality and cultural backgrounds. Finally, we have provided a set of recommendations for the design of future technological solutions to reduce the negative impact of social media by supporting different approaches to content moderation.

## Supplementary Information

Below is the link to the electronic supplementary material.

Supplementary material 1.

Supplementary material 2.

## Data Availability

No datasets were generated or analysed during the current study.

## Abbreviations

ED: Eating disorders
AI: Artificial intelligence
