# Supplementary material for: Thigh gaps and filtered snaps: a qualitative study exploring opportunities to mitigate social media harm through content moderation for people with eating disorders
Source: J Eat Disord. 2026 Jan 21;14:61. doi: 10.1186/s40337-025-01504-7 (PMC12998281; doi:10.1186/s40337-025-01504-7)
Supplement: Supplementary file 1 — Supplementary material 1. [file 40337_2025_1504_MOESM1_ESM.docx]

**Focus group with experts by lived experience**

Attendees: Lived experience X 18 (5 foucs groups)

Type: Online

Duration: 90 minutes

**Aims:**

1. Understand the role of social media (positive and negative) in different stages of eating disorders (early, late and recovery).
2. Understand the requirement of individuals with lived experience in terms of safety in social media environment.
3. Understand the perception of individuals with lived experience regarding AI and usage of AI, and potential solutions.
4. Exploring the characteristics of harmful, safe and ambiguous social media content for different categories identified from the interview.

**Outcome:**

- To develop a set of moderation guidelines in terms of harmful content for body image and eating disorders
- To understand their perception regarding usage of technology and AI in mitigating harm from social media space.

**Introduction to the program:**

- Background of the project
- The proposed aim and purpose of the project
- The aim of today’s lived experience focus group
- Verbal consent
- House-keeping

**Icebreaker**

- Introduce yourself and tell us your favourite social media platform and why? (Go around the Zoom room)

**A. AREA OF DISCUSSION: Role of social media in different stages of eating disorders (20 mins)**

**Role of social media in different stages of eating disorder [Whole group discussion]**

What role does social media play in:

- Early stage of eating disorder
- Late stage of eating disorder
- During recovery

**B. AREA OF DISCUSSION: Requirement for a safer social media environment (15 mins)**

**Manifesto creation [breakout room]**

- You are writing a manifesto for the government to advocate for safer and more supportive changes on social media platforms for people with (or at risk of) eating disorders.
- Brainstorm 'we need' statements, and then as a group select the final three 'we need' statements that highlight the most important points.

**C. AREA OF DISCUSSION: Perceptions regarding Artificial Intelligence (AI) and potential solutions (20 mins)**

**Government has created Pikachu AI**

- The government has listened to your manifesto and has agreed to embed an Artificial Intelligence (AI) named Pikachu AI into the social media platform.
- AI can do things such as learning, reasoning and problem-solving. It's a way of making computers think and act like people.
- Now, what Pikachu AI can do to make social media a safer place is up to you. You can decide what it can do.
- What does Pikachu AI do?
- How does Pikachu AI make you feel?
- How is it making people's lives easier?
- What are the potential challenges?
- Whis is the biggest advocate of Pikachu AI? Why?
- Who is the biggest critic of Pikachu AI? Why?

**D. AREA OF DISCUSSION: Characteristics of harmful and safe social media content (20 mins)**

**Explore the rules for Pikachu AI: [Breakout room]**

- The Pikachu AI needs few rules to make it work better while looking at certain contents in social media related to body image and eating disorder. There are 8 themes, ask the participants to select contents that would be under the "Must not show" rules and "Permitted to show" rules for each of these themes.
- Themes:
- Weight loss content/ content influencing changes to body shape/size
- Weight loss or performance-enhancing drugs
- Eating disorder recovery
- Cosmetic surgery or other minimally invasive cosmetic procedures
- Beauty tutorials
- Food and nutrition
- Exercise
- Body checking (creator is checking their body either in the camera, on weighing scales or by measuring parts of their body)

**E. AREA OF DISCUSSION: Conclusion**

- Any last questions or thoughts on tonight’s focus group?
- Next steps
