# Supplementary material for: Thigh gaps and filtered snaps: a qualitative study exploring opportunities to mitigate social media harm through content moderation for people with eating disorders
Source: J Eat Disord. 2026 Jan 21;14:61. doi: 10.1186/s40337-025-01504-7 (PMC12998281; doi:10.1186/s40337-025-01504-7)
Supplement: Supplementary file 2 — Supplementary material 2. [file 40337_2025_1504_MOESM2_ESM.docx]

**Interview Schedule:**

Attendees: Researchers X12

Type: Online

Duration: 60 minutes

**Aims:**

1. Understand the role of social media in body image, its use in promotion of positive and negative body image.
2. Understand the role of diversity in the populations that researchers and clinicians engage with.
3. Identifying the characteristics of harmful, safe and ambiguous social media content.
4. Explore the opportunities in social media intervention space.

**Outcome:**

- To develop a set of moderation guidelines in terms of harmful content for body image and eating disorders
- To refine a set of topics to take into the next phase of design

**Can you tell me a little about yourself? (5 minutes)**

- Can you introduce yourself:
- Who you are?
- Where do you work?
- What do you do?
- Can you tell me a little about your work in the body image and eating disorders space?
- How many years have you been working in this space?

**A. AREA OF DISCUSSION: Understand the role of social media in body image and eating disorders (15 minutes)**

**Questions**

**Negative impact of social media on body image**

1. What are your thoughts on the negative impact of social media on body image?

- **Prompt:** What are some of the trends or patterns in how social media has negatively influenced body image?
- **Prompt:** What do you think the differences are between different platforms like Instagram, Twitter and TikTok?

**If they are working in the social media space,**

- **Prompt:** Can you discuss any examples from your own work that highlight the link between social media and negative body image?

**Challenges and opportunities to reduce the negative impact of social media**

1. How can we as researchers/clinicians and public help to reduce the social media impact on negative body image?

- **Prompt:** What strategies or interventions can be implemented to address the negative impact on body image?
- **Prompt:** At present, what are your thoughts on the most pressing challenges in addressing the negative impact of social media on body image and how can we address them?
- **Prompt:** How can we use social media itself to mitigate some of its negative impact?

**Positive impact of social media**

1. How can social media platforms themselves contribute to fostering a more positive body image?
2. Can you think about any social media accounts or trends that have used social media to foster positive body image?

**B. AREA OF DISCUSSION: Diversity in research and its importance (15 minutes)**

**Questions**

**Understanding of underrepresented group in body image space**

1. In your view, who are the underrepresented groups in the body image and eating disorder space? (prompt: culture, age, gender)- specific challenges
2. Have you worked with any of these underrepresented groups?

**Understanding of their views on working with under-represented group**

If **YES**

- **Prompt:** What do you think are the barriers when working with these groups?
- **Prompt:** Is there anything we can do to improve representation in research of social media more broadly? **(Addressing challenges)**

If NO

- **Prompt:** Is there anything we can do to improve representation in research of social media?

**C. AREA OF DISCUSSION: Identifying the characteristics of harmful, safe and ambiguous social media content (20 minutes)**

**Questions**

**Harmful social media content**

1. How would you define harmful social media content for body image and eating disorders? **(characteristics of harmful content)**

- **Prompt:** What kind of social media content could be considered as harmful?
- **Prompt:** You talked lot about images/ videos, what role do you think the captions, hashtags and comments have in contributing to the harmfulness?

1. [If they have talked about it] A lot of harmful content you discussed seems like it might be harmful to someone with anorexia for example, do you think this is different for people with EDs in larger bodies? **(difference in impact across different body sizes)**

**Safe social media content**

1. How would you define safe social media?

- **Prompt:** What do you think about the body-positive and body-neutral content?
- **Prompt:** How would you categorise a healthy food suggestion content?

**Ambiguous social media content**

1. Is there anything that you think is ambiguous or difficult to define as safe or harmful?

**D. AREA OF DISCUSSION: Potential solutions (10 minutes)**

**Blue sky social media intervention**

1. If money was not an issue, what tool would you create for improving body image?

- **Prompt:** Who would this be most beneficial for?
- **Prompt:** What would having this type of solution achieve?
- **Prompt:** What would be the challenges of making this idea a reality?
- **Prompt:** How would these solutions change across underrepresented groups, if at all?

**Views on trained computer (automation)**

1. Are there any aspects of interventions for body image that you would want to be automated so that a computer can do the tasks (this might be things you find boring for example, that take a lot of time, or that you find difficult to do)?

- **Prompt:** Are there any specific ethical considerations that need to be taken into account?
- **Prompt:** What are the scenarios where you would absolutely need a human to be involved?
- **Prompt:** Imagine, we had a team of volunteers who were willing to scan through social media and find harmful social media content. Who do you think the ideal volunteer would be? Why?

**E. AREA OF DISCUSSION: Closing**

This is all I wanted to ask from my side. Thank you very much for your time! I really

appreciate your contribution and I found all the things you said really interesting.

**Questions**

1. Is there anything else that you would like to add or comment on?
2. Do you have any other observations about how stakeholders may feel about technologies like ours?

If yes – follow up participant’s thoughts and possibly ask further

If no – Thank you very much for your time!
